# Functional Mapping of Phenotypic Plasticity of *Staphylococcus aureus* Under Vancomycin Pressure

**DOI:** 10.3389/fmicb.2021.696730

**Published:** 2021-09-09

**Authors:** Dengcheng Yang, Xuyang Zheng, Libo Jiang, Meixia Ye, Xiaoqing He, Yi Jin, Rongling Wu

**Affiliations:** ^1^Center for Computational Biology, College of Biological Sciences and Biotechnology, Beijing Forestry University, Beijing, China; ^2^College of Biological Sciences and Biotechnology, Beijing Forestry University, Beijing, China; ^3^Department of Public Health Sciences and Statistics, Center for Statistical Genetics, The Pennsylvania State University, Hershey, PA, United States

**Keywords:** phenotypic plasticity, *Staphylococcus aureus*, functional mapping, vancomycin, growth curve

## Abstract

Phenotypic plasticity is the exhibition of various phenotypic traits produced by a single genotype in response to environmental changes, enabling organisms to adapt to environmental changes by maintaining growth and reproduction. Despite its significance in evolutionary studies, we still know little about the genetic control of phenotypic plasticity. In this study, we designed and conducted a genome-wide association study (GWAS) to reveal genetic architecture of how *Staphylococcus aureus* strains respond to increasing concentrations of vancomycin (0, 2, 4, and 6 μg/mL) in a time course. We implemented functional mapping, a dynamic model for genetic mapping using longitudinal data, to map specific loci that mediate the growth trajectories of abundance of vancomycin-exposed *S. aureus* strains. 78 significant single nucleotide polymorphisms were identified following analysis of the whole growth and development process, and seven genes might play a pivotal role in governing phenotypic plasticity to the pressure of vancomycin. These seven genes, SAOUHSC_00020 (*walR*), SAOUHSC_00176, SAOUHSC_00544 (*sdrC*), SAOUHSC_02998, SAOUHSC_00025, SAOUHSC_00169, and SAOUHSC_02023, were found to help *S. aureus* regulate antibiotic pressure. Our dynamic gene mapping technique provides a tool for dissecting the phenotypic plasticity mechanisms of *S. aureus* under vancomycin pressure, emphasizing the feasibility and potential of functional mapping in the study of bacterial phenotypic plasticity.

## Introduction

Phenotypic plasticity is the capacity of an individual genotype to produce different phenotypes (e.g., morphology or behavior) in specific environments (Anderson et al., 2012; Ashander et al., 2016; Kelly, 2019); phenotypic plasticity is a manifestation of biological adaptability and is the main mechanism by which animals and plants respond to some environmental change (Botero et al., 2015; Pozo et al., 2015; Bonamour et al., 2019). A vast number of studies have investigated phenotypic plasticity in plants and animals; however, comparatively few reports have examined the phenotypic plasticity of microorganisms (Savvides et al., 2017). The response mechanisms of microorganisms to environmental change are highly complex. Phenotypic plasticity can mitigate the effects of environmental changes on microbial growth, heredity and development (Moeller and Sanders, 2020). Bacteria show great adaptability to new pressure in changing environments (Sagiv et al., 2015; van Opijnen et al., 2016; Ospina-Serna et al., 2020). Therefore, studying the phenotypic plasticity of microorganisms is key to understanding the response mechanisms of microorganisms to environmental changes.

Genome-wide association studies (GWAS) are an important tool for evaluating phenotypic plasticity. Since the publication of the first successful human GWAS in 2005 (Klein et al., 2005), GWAS have become an increasingly important research method for geneticists. The GWAS method has enabled the characterization of complex diseases and the identification of common genetic variations associated with particular characteristics. However, genetic variation manifests differently in bacteria compared with in humans and other polyploid organisms. Homologous recombination and chromosome segregation occur during human reproduction. On the other hand, bacteria are haploid and asexual with highly structured populations in which horizontal gene transfer and recurrent mutations occur (Read and Massey, 2014). As a result, the development and adaptation of GWAS for use in microorganisms has been relatively slow (Buchanan et al., 2017), although there are some examples of its successful application. GWAS have successfully been applied to identify mutations in the RNA polymerase rpoB gene of *Staphylococcus aureus* that are associated with drug resistance phenotypes (Alam et al., 2014) and to explore the interactions between *Escherichia coli* and *S. aureus* genes (He et al., 2017).

The majority of phenotypic plasticity studies using the GWAS method so far have modeled static phenotypic data measured at a single point in time (Gan et al., 2019). Phenotypes are developmental and changes over time; however, GWAS can only be used to identify phenotypic correlations at a specific time and quantitative trait locus (QTL), without considering dynamic phenotypic development information (Wang et al., 2014; Jiang et al., 2019). Therefore, to assess developmental phenotypic plasticity, dynamic modeling of a series of phenotypes measured over a period of time is required (Li and Wu, 2010). Functional mapping takes into account the biological mechanisms and dynamic processes of phenotypic formation; in this technique, phenotypic traits are continuously measured over a series of time points, and biologically meaningful information such as growth curves is employed to fit these observations. These data are then used to establish a hybrid model under various genetic designs, and the maximum likelihood method and expectation–maximization algorithms are used to estimate parameters. Likelihood ratio statistics have been applied to determine the loci of phenotypic plasticity genes and to quantify their dynamic changes using temporal and spatial scales (Ma et al., 2002; Wu and Lin, 2006). Functional mapping is an accurate and powerful tool for determining the QTL of key growth traits in various species (Li and Sillanpaa, 2015). Bivariate functional mapping is a technique that integrates bivariate data from different conditions into a functional map framework, revealing the expression patterns of phenotypic plasticity genes in different environments (Zhao et al., 2005).

*S. aureus* is a clinically important bacterial species, which is effectively treated using the antibiotic vancomycin (Shahin et al., 2020; Simon et al., 2020). However, with the continuous use of antibiotics, vancomycin-resistant *S. aureus* strains are increasingly developing that can adapt to antibiotic environments and show strong phenotypic plasticity (Bakri et al., 2007). In this study, functional mapping and bivariate functional mapping models were applied to evaluate microbial phenotypic plasticity for the first time. We monitored the growth status of *S. aureus* under four vancomycin concentrations and used these data to establish a model framework with which to study the phenotypic plasticity of microbial development using functional mapping and multivariate analyses. Our model revealed the gene loci and mechanisms related to phenotypic plasticity in *S. aureus* and for general applications in the study of phenotypic plasticity in microorganisms.

## Materials and Methods

### Bacterial Strains and Pre-cultivation

First, we measured the vancomycin susceptibilities of 99 vancomycin-sensitive *S. aureus* strains by subjecting them to vancomycin treatment. Forty-one of the strains were subjected to vancomycin treatment in our previous study (Rong et al., 2019). With the exception of strain S10’, the *S. aureus* strains showed varying degrees of resistance to vancomycin. Another 58 vancomycin-sensitive strains (S47−S105, with the exception of S86) were clinical isolates from Beijing Chaoyang Hospital and incubated on brain heart infusion (BHI) (Oxoid, Basingstoke, United Kingdom) agar plates supplemented with vancomycin (Sigma-Aldrich, St. Louis, MO, United States) at 50% of the initial minimum inhibitory concentration (MIC) at 37°C. The strains were transferred to fresh medium with the same vancomycin concentration every 24 h for 4 days. Following treatment, we re-calculated the MIC of vancomycin for each strain every 4 days for a total of 60 days, as described previously (Wang et al., 2017). At the end of the treatment, we determined the final MIC value for each strain using the *E*-test method (bioMérieux); 58 of the strains showed varying degrees of vancomycin resistance. The backgrounds and vancomycin susceptibilities of all strains are listed in Supplementary Table 1. Before the plasticity experiment, single colonies were transferred to BHI medium and shaken overnight at 37°C.

### Growth Phenotypic Plasticity Assay

Ninety-nine strains of *S. aureus* were inoculated into fresh BHI medium supplemented with 0, 2, 4 or 6 μg/mL vancomycin to an initial cellular concentration of 6 × 10^3^ CFU/mL. Growth curve experiments were carried out in 96-well plates (Corning, New York, NY, United States) containing 150 μl volumes in triplicate. Bacterial liquid concentrations were determined by measuring the optical density at 600 nm at 14 time points (1, 2, 4, 6, 8, 10, 12, 16, 20, 24, 30, 36, 42, and 48 h) over a 48 h period. Then, three growth curve equations, Gompertz, Logistic, and Richards, were used to fit the *S. aureus* developmental phenotype data (Equation 1), and the most suitable equation was selected by least square method. In the following equation, g(T) represents the growth of the strain at time t, A represents the growth degree, R represents the maximum specific growth rate, λ represents the time of lag phase ends.

(1) $$g\,\left(t\right)=\left\{ \begin{array}{cc} A\cdot \exp\left\{-\exp\left[\frac{\mathrm{R\cdot e}}{A}\,\left(\lambda -t\right)+1\right]\right\}\;\text{Gompertz} & \\ \frac{A}{1+\exp\left[\frac{4\,R}{A}\,\left(\lambda -t\right)+2\right]}\;\text{Logistic} & \\ A\{1+s\cdot \exp\left(1+s\right)\cdot \exp & \\ \left[\frac{R}{A}{\left(1+s\right)}^{\left(1+\frac{1}{s}\right)}\left(\lambda -t\right)\right]\}{}^{\left(-\frac{1}{s}\right)}\text{Richards} & \end{array}\right.$$

### Whole Genome Sequencing

Genomic DNA was extracted using the TIANamp Bacteria DNA Kit (Tiangen, Beijing, China) according to the manufacturer’s protocol. In total, 99 *S. aureus* strains were genotyped by genome sequencing on the Illumina HiSeq 4000 platform (Illumina Inc., San Diego, CA, United States) at Allwegene (Beijing, China). Genome detection, database construction and sequencing, among other factors, may have affected the quality and quantity of the data and therefore affected the results of downstream analyses. To acquire high-quality sequencing data, we performed several quality control steps in the original data, such as removing low-quality reads and joints and calculating the sequencing error rate, Q20 and Q30 statistics, and GC content. *S. aureus* subspecies NCTC8325 (NC_007795.1) was selected as the control strain for comparison; the comparison results were acquired in BAM format. SAM tools (Li et al., 2009) were used to organize the results, mark repetitive sequences and filter the resulting single nucleotide polymorphisms (SNPs).

Sequencing libraries were constructed using inserted fragment sizes of 300 and 150 bp were sequenced at both ends of the library via paired-end sequencing. The original data size was 697.937200−2658.411600 Mb. After quality control, the data range was 697.16−2581.08 Mb. A total of 110,675 SNPs were obtained from the whole genome data of 99 *S. aureus* strains following data processing. The sequencing statistics, including the average sequencing data quality and comparisons with the reference sequence, are shown in Supplementary Tables 2, 3. All sequencing data were deposited in NCBI repository^1^ and the serial numbers are listed in Supplementary Table 1.

### Functional Mapping Model

Functional mapping integrates the mathematical relationships between different traits or variables within a genetic mapping framework, where it applies universal biological laws to model the genotypic effects of QTLs. In this study, a functional mapping model was applied to identify key genes affecting the growth of *S. aureus* at various vancomycin concentrations. Population structure effects were corrected using FastStructure software (Raj et al., 2014), which could be divided into eight groups.

#### Statistical Model

The phenotype of the *j*th genotype of a QTL was assumed to fall within a multivariate normal distribution:

(2) $$f_{j}\,\left(y\right)=\frac{1}{{\left(2\,\pi \right)}^{\frac{T}{2}}\,{\left|\Sigma \right|}^{\frac{1}{2}}}\,\exp\left[-\frac{1}{2}\,\left(y-g_{j}\right)\,\Sigma^{-1}\,{\left(y-g_{j}\right)}^{T}\right]$$

Here **g**_*j*_ was fitted using Equation 1, and the best-fitting equation was selected using the least square method. The fitted mean vector is shown in Equation 3:

(3) $${\text{g}}_{j}=\left(g_{j}\,\left(1\right),g_{j}\,\left(2\right)\,\cdots \,g_{j}\,\left(T\right)\right)$$

The first-order autoregressive (AR[1]) (Yap et al., 2009) was applied to obtain the **Σ** value. This model comprised the two parameters ρ and σ^2^:

(4) $$\Sigma =\sigma^{2}\,\left[\begin{array}{cccc} 1 & \rho & \cdots & \rho^{m-1}\\ \rho & 1 & \cdots & \rho^{m-2}\\ \cdots & \cdots & \cdots & \cdots \\ \rho^{m-1} & \rho^{m-2} & \cdots & 1 \end{array}\right]$$

The likelihood function was then constructed as follows:

(5) $$L\,\left(\Omega \right)=\prod_{i=1}^{N}\left[\sum_{j=1}^{2}p_{i\,j}\,f_{j}\,\left(y_{i}\right)\right]$$

#### Hypothesis Tests

Evaluating significant QTL was conducted by hypothesis tests:

*H_0_:(A_1_,R_1_,λ_1_,S_1_) ≡ (A_2_,R_2_,λ_2_,S_2_) for j = 1,…,J*
*H_1_: Not all equalities above do not hold*

Hypothesis test statistics were calculated using the log-likelihood ratio (LR) (Churchill and Doerge, 1994).

(6) $$\text{LR}=-2\,\log\left[\frac{L\,\left(\tilde{\Omega }\right)}{L\,\left(\hat{\Omega }\right)}\right]$$

where $\tilde{\Omega }$ and $\hat{\Omega }$ represent the maximum likelihood estimates under *H*_0_ and *H*_1_, respectively. The threshold was determined using the permutation test method, in which 1,000 repeated arrangements were made, and the relationship between genotype and bivariate phenotype was randomly reshuffled in each arrangement. A minimum *P*-value from each arrangement was selected to construct an incremental vector comprising 1,000 elements. Significance was determined at *P* < 0.05, and the 50th value was taken as the threshold.

### Bivariate Functional Mapping Model

A structured antedependence (SAD) model was applied to approximate time correlation covariance matrices of longitudinal traits based on functional mapping. Here, we established a functional mapping method for multiple longitudinal traits. This method can integrate and analyze multiple shapes by applying functional mapping. SAD models are useful tools for designing effective early selection programs for animal and plant breeding, identifying genes that control the progress of human diseases, and raising and solving biological problems at the interface of genetics, development and evolution (Zhao et al., 2005; Wang et al., 2019). The effect of population structure was corrected using FastStructure software (Raj et al., 2014).

#### Statistical Model

The likelihood function of bivariate data of N individuals affected by J genes was formulated by

(7) $$L\,\left(\Omega \right)=\prod_{i=1}^{N}\left[w_{1\,i}\,f_{1}\,\left(z_{i}\right)+w_{2\,i}\,f_{2}\,\left(z_{i}\right)\right]$$

where *w*_1_ and *w*_2_ represent the conditional probabilities of genotypes, and *f*_*j*_(**z**_*i*_) represents the corresponding multivariate normal distribution.

(8) $$f_{j}\,\left(y\right)=\frac{1}{{\left(2\,\pi \right)}^{\frac{T}{2}}\,{\left|\Sigma \right|}^{\frac{1}{2}}}\,\exp\left[-\frac{1}{2}\,\left(z-g_{j}\right)\,\Sigma^{-1}\,{\left(z-g_{j}\right)}^{T}\right]$$

Here, **g**_*j*_ represents a bivariate mean vector, which was obtained by fitting various processing data from Equation 1.

(9) $$g_{j}=\left(g_{1\,j}\,\left(1\right),g_{2\,j}\,\left(1\right)\,\cdots \,g_{1\,j}\,\left(T\right),g_{2\,j}\,\left(T\right)\right)$$

Then, a first-order SAD (1) model was applied to model **Σ**.

(10) Σ=(ΣxΣy⁢x⁢Σx⁢yΣy)

where **Σ_x_** and **Σ_y_** represent covariance matrices of bivariate data, respectively, and **Σ**_**x***y*_ and **Σ_y_**_*x*_ represent covariance matrices between two sets of data.

#### Hypothesis Tests

Evaluating significant QTL was conducted by hypothesis tests:

*H_0_: **Ω**_1_ ≡ **Ω**_2_ for j = 1,…,J*
*H_1_: Not all equalities above do not hold*

where **Ω** is the set of all parameters comprising the mean curve. The hypothesis test statistics were calculated via LR, and the threshold was calculated using a permutation test.

### Genotype–Phenotype Variation Analysis

Based on the functional positioning and binary curve analyses, the curve parameters fitted by the growth curve equation of different strains were determined. The parameters in this model are as follows: λ Represents the time of the lag phase ends, R represents the maximum specific growth rate of the growth curve, and A represents the growth degree (Jiang et al., 2015). The three growth parameters obtained were biologically significant. The growth curve parameters were compared using *t*-tests to further explore the significance of the QTLs and how they affect the phenotypic plasticity of *S. aureus* in relation to growth.

## Results

### Growth Curve Assay

After 60 days vancomycin treatment, the MICs of most strains were increased. The backgrounds of vancomycin treated strains (S1–S105) and parental strains (S_*p*_1–S_*p*_105) are listed in Supplementary Table 1. All subsequent studies were performed on the vancomycin treated strains, including whole genome sequencing.

**FIGURE 1:**
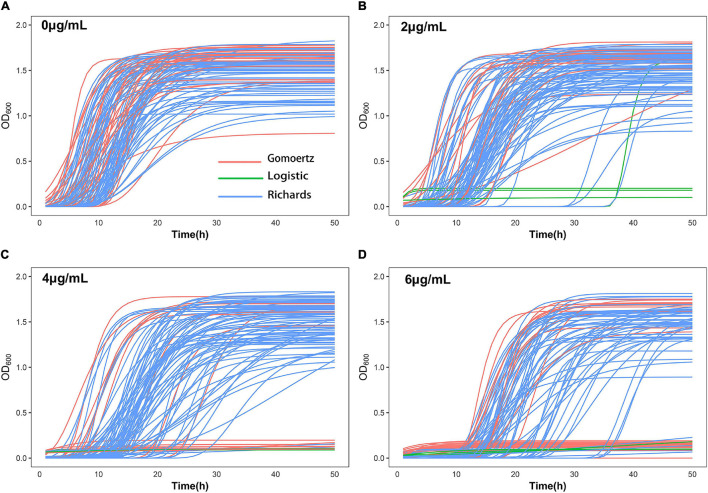
Growth curve fitting of 99 *S. aureus* strains. The growth curves of all strains at 0 μg/mL **(A)**, 2 μg/mL **(B)**, 4 μg/mL **(C)**, and 6 μg/mL **(D)** vancomycin treatment were fitted by Gompertz, Logistic or Richards equation. Each line represents the growth of a strain, red represents Gompertz equation, blue represents Richards equation, and green represents Logistic equation.

The growth curve equation was used to fit the growth data of the 99 *S. aureus* strains grown in increasing concentrations of vancomycin (0, 2, 4, and 6 μg/mL) and the least square method was used to select the best fitting equation for each individual (Figure 1). The growth curves differed between the strains, which is likely due to their varying MICs and degrees of adaptability to vancomycin treatment. In the absence of antibiotics, all 99 *S. aureus* strains exhibited typical growth curves (Figure 1A). At a vancomycin concentration of 2 μg/mL, three strains (S22, S25, and S96) were not able to grow. Furthermore, the lag times of 32 strains (S2, S4, S6, S12, S14, S18, S20, S21, S24, S26, S30, S33, S36, S38, S42, S49, S50, S51, S54, S56, S57, S61, S62, S76, S80, S83, S93, S94, S95, S97, S104, and S105) were significantly delayed, and the time to reach the logarithmic growth phase was longer than that without antibiotic supplementation (Figure 1B). At a vancomycin concentration of 4 μg/mL, 18 strains (S1, S2, S4, S6, S12, S14, S17, S20, S22, S24, S25, S33, S36, S42, S49, S54, S95, and S96) did not grow, and the lag times and stationary phases of 35 strains were delayed (Figure 1C). Thirty-two strains (S1, S2, S4, S5, S6, S7, S12, S14, S17, S19, S20, S21, S22, S24, S25, S26, S29, S30, S31, S33, S36, S42, S49, S54, S62, S77, S88, S91, S94, S95, S96, and S103) did not grow at 6 μg/mL group due to the high vancomycin concentration. For each strain, differences between four growth curves under different vancomycin concentrations could directly reflect growth phenotypic plasticity (Figure 1). After equation fitting, three key parameters (A, R, and λ) were collected (Supplementary Table 4). The curve equations could associate with gene data and locate the SNPs that affect the dynamic growth process by functional mapping.

### Functional Mapping

**FIGURE 2:**
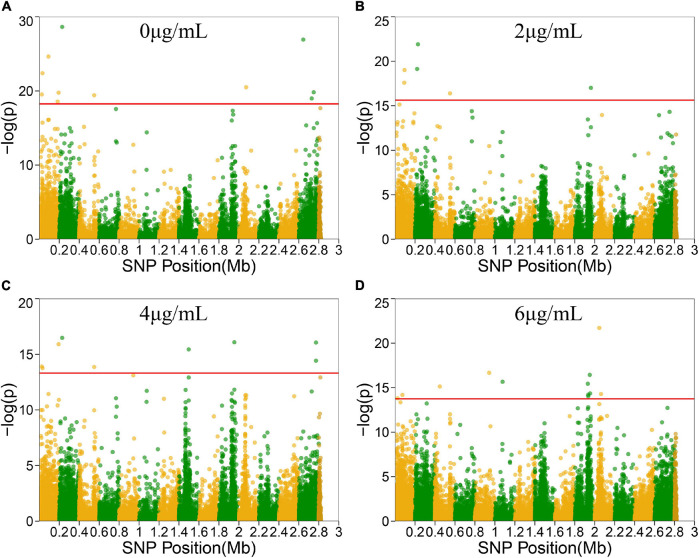
Manhattan plots of functional mapping. Manhattan plot of the significance test based on log-likelihood ratio (LR) for associations between the phenotypic growth differences in different groups and SNPs distributed on the *S. aureus* genome. SNPs positions in genome were divided by two colors. Each dot in the plot represented an SNP, and a reference line was used on the *y*-axis to reflect genome-wide significance. **(A–D)** Represent Manhattan plots of four treatment groups.

To locate candidate genes related to *S. aureus* resistance development and phenotypic plasticity at different vancomycin concentrations, the dynamic growth data of 99 *S. aureus* strains were analyzed by functional mapping. Thirty-eight significant SNPs were identified under the four vancomycin concentrations (Figure 2), and their gene locations were functionally annotated, including 16 missense mutations and 16 synonymous mutations (Table 1).

**TABLE 1:** Gene annotation of significant sites in functional mapping.

Vancomycin concentration	Position	–log*P*-value	aa_mutate	Gene ID	Annotion
0 μg/mL	25,261	19.52	A < - > T	SAOUHSC_00020	Transcriptional regulatory protein WalR
0 μg/mL	31,826	22.39	G < - > G	SAOUHSC_00025	Hypothetical protein
0 μg/mL	92,210	24.64	P < - > P	SAOUHSC_00085	Hypothetical protein
0 μg/mL	183,485	18.55	Y < - > Y	SAOUHSC_00169	Peptide ABC transporter permease
0 μg/mL	193,712	19.76	N < - > S	SAOUHSC_00176	Extracellular solute-binding protein
0 μg/mL	229,384	28.63	/	/	Non-coding region
0 μg/mL	550,323	19.41	N < - > S	SAOUHSC_00544	Fibrinogen-binding protein SdrC
0 μg/mL	2,072,412	20.49	/	/	Non-coding region
0 μg/mL	2,644,158	26.92	F < - > F	SAOUHSC_02871	Hypothetical protein
0 μg/mL	2,728,755	18.98	V < - > I	SAOUHSC_02967	Arginine/ornithine antiporter
0 μg/mL	2,748,895	19.82	T < - > S	SAOUHSC_02982	Hypothetical protein
2 μg/mL	92,210	17.59	P < - > P	SAOUHSC_00085	Hypothetical protein
2 μg/mL	94,818	19.01	C < - > C	SAOUHSC_00089	Hypothetical protein
2 μg/mL	220,967	19.13	I < - > V	SAOUHSC_00199	Acyl CoA:acetate/3-ketoacid CoA transferase
2 μg/mL	229,384	21.90	/	/	Non-coding region
2 μg/mL	550,323	16.39	N < - > S	SAOUHSC_00544	Fibrinogen-binding protein SdrC
2 μg/mL	1,961,488	17.01	A < - > T	SAOUHSC_02078	Phi PV83 orf 10-like protein
4 μg/mL	25,261	13.90	A < - > T	SAOUHSC_00020	Transcriptional regulatory protein WalR
4 μg/mL	32,318	13.76	N < - > N	SAOUHSC_00025	Hypothetical protein
4 μg/mL	193,712	15.91	N < - > S	SAOUHSC_00176	Extracellular solute-binding protein
4 μg/mL	229,384	16.48	/	/	Non-coding region
4 μg/mL	550,323	13.86	N < - > S	SAOUHSC_00544	Fibrinogen-binding protein SdrC
4 μg/mL	1,496,942	15.44	M < - > L	SAOUHSC_01560	Hypothetical protein
4 μg/mL	1,954,079	16.08	Y < - > F	SAOUHSC_02063	PV83 orf 23-like protein
4 μg/mL	2,771,775	14.42	L < - > F	SAOUHSC_02998	Capsular polysaccharide biosynthesis protein Cap5C
4 μg/mL	2,771,777	16.05	L < - > L	SAOUHSC_02998	Capsular polysaccharide biosynthesis protein Cap5C
6 μg/mL	25,261	13.81	A < - > T	SAOUHSC_00020	Transcriptional regulatory protein WalR
6 μg/mL	73,870	14.18	D < - > D	SAOUHSC_00069	Immunoglobulin G-binding protein A
6 μg/mL	448,470	15.13	/	/	Non-coding region
6 μg/mL	942,441	16.67	/	/	Non-coding region
6 μg/mL	1,076,587	15.66	R < - > R	SAOUHSC_01121	Alpha-hemolysin
6 μg/mL	1,930,634	15.44	N < - > N	SAOUHSC_02026	Phi ETA orf 58-like protein
6 μg/mL	1,934,358	14.04	F < - > F	SAOUHSC_02029	Phi ETA orf 56-like protein
6 μg/mL	1,935,188	14.21	T < - > T	SAOUHSC_02030	Phi ETA orf 55-like protein
6 μg/mL	1,950,139	16.44	H < - > H	SAOUHSC_02050	Terminase small subunit
6 μg/mL	1,953,238	14.36	I < - > I	SAOUHSC_02060	Phi PVL orf 51-like protein
6 μg/mL	2,045,672	21.71	K < - > K	SAOUHSC_02182	Tail length tape measure protein
6 μg/mL	2,062,915	14.28	R < - > S	SAOUHSC_02214	Hypothetical protein

SNP 25261 is located within the SAOUHSC_00020 gene, which is related to the transcriptional regulatory protein WalR. SAOUHSC_00169 (containing SNP 183485) encodes the ABC transporter permease, which is involved in the transport of various molecules such as amino acids, lipids, lipopolysaccharides, inorganic ions, peptides and sugars; this transporter may play a crucial role in bacterial drug resistance. SNP 193712 is located within SAOUHSC_00176, a chemoreceptor that recognizes constituents of transport systems and initiates signal transduction pathways. SAOUHSC_00544 (harboring SNP 550323) encodes fibrinogen-binding protein SdrC. SNP 2728755 is located in SAOUHSC_02967, which is involved in the formation of biofilms, arginine synthesis and pH regulation. SAOUHSC_00199 (containing SNP 220967) encodes acyl-CoA, which also plays an important role in the formation of *S. aureus* biofilms. SNP 1961488 is located in SAOUHSC_02078, which encodes phi PV83 orf 10-like protein. SNP 2771775 is located in SAOUHSC_02998, which is involved in the synthesis of the outer capsule of the cell wall. SAOUHSC_00069 (containing SNP 73870) encodes immunoglobulin G-binding protein A, which plays an important role in the inhibition of the host innate and adaptive immune responses. SAOUHSC_02026 (containing SNP 1930634), SAOUHSC_02029 (containing SNP 19304358), SAOUHSC_02030 (containing SNP 1935188), and SAOUHSC_02060 (containing SNP 1953238) encode phi ETA orf 58-like protein, phi ETA orf 56-like protein, phi ETA orf 55-like protein and phi PVL orf 51-like protein, respectively. The SAOUHSC_01121 gene (containing SNP 1076587) is related to alpha-hemolysin, which binds to the membrane of eukaryotic cells, resulting in the release of low-molecular weight molecules, eventually leading to osmotic lysis. Alpha-hemolysin also inhibits host neutrophil chemotaxis to the bacterial lesion. SAOUHSC_02182 (containing SNP 2045672) encodes the tail tape measure protein. The SNPs at positions 229384, 2072412, 448470, and 942441 were located within non-coding regions.

### Bivariate Functional Mapping

**FIGURE 3:**
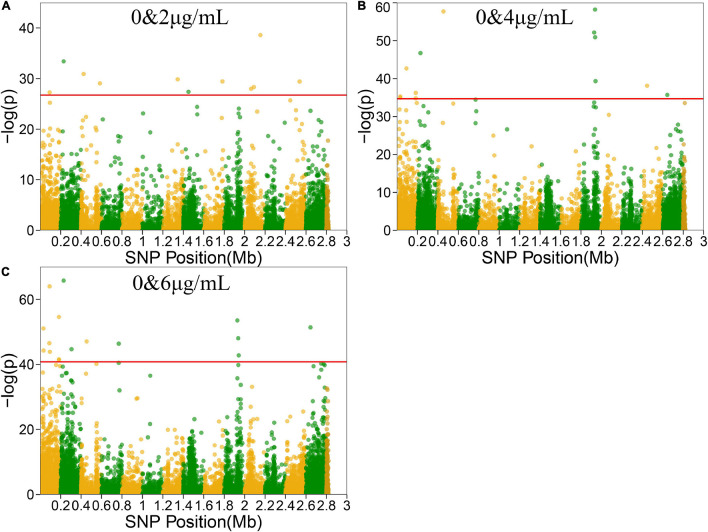
Manhattan plots of bivariate functional mapping. Manhattan plot of the significance test based on the associations between phenotypic plasticity of microbial growth in the environment with or without antibiotics and SNPs. Each dot in the plot represented an SNP, and a reference line was used on the *y*-axis to reflect genome-wide significance. **(A–C)** Bivariate analysis of Manhattan plots with 0 and 2 μg/mL, 0 and 4 μg/mL, or 0 and 6 μg/mL vancomycin treatment.

Functional mapping focuses on the genes that affect the dynamic growth of strains in different concentrations of antibiotics using univariate phenotype data. Growth curves in the presence or absence of antibiotics formed the typical bivariate phenotype, while bivariate functional mapping can simultaneously analyze correlated phenotypes in plasticity study. The differences in the growth curves of *S. aureus* cultured with and without vancomycin directly reflected the phenotypic plasticity of *S. aureus*. Therefore, we performed bivariate functional mapping to analyze binary data collected following *S. aureus* growth at vancomycin concentrations of 0 and 2 μg/mL, 0 and 4 μg/mL, and 0 and 6 μg/mL (Figure 3). A total of 40 significant SNP sites were found, including 6 missense mutations and 26 synonymous mutations (Table 2). The SNP at position 2062927 is located within the SAOUHSC_02214 gene, which encodes a conserved hypothetical phage protein. SAOUHSC_00025 (containing SNP at site 31826) encodes an uncharacterized GRAM_POS_ANCHORING domain-containing protein. SNP 183439 is located in SAOUHSC_00169, which encodes the same permease transporter protein discussed above. The SNP at position 1928590 is located in SAOUHSC_02023, an important player in cell separation, daughter cell formation and autolysis. The SAOUHSC_02033 gene (containing SNP 1938367) is related to the phage tape measure protein. The SNP at position 1942424 is located in SAOUHSC_02036, which encodes a phage structural protein. The SAOUHSC_02871 gene (containing SNP 2644158) encodes an acetyltransferase.

**TABLE 2:** Gene annotation of significant sites in bivariate functional mapping.

Vancomycin concentration	Position	–log*P*-value	aa_mutate	Gene ID	Annotion
0 and 2 μg/mL	92,210	27.26615386	P < - > P	SAOUHSC_00085	Hypothetical protein
0 and 2 μg/mL	229,384	33.40197554	/	/	Non-coding region
0 and 2 μg/mL	424,511	30.89808354	/	/	Non-coding region
0 and 2 μg/mL	584,033	29.04832498	D < - > D	SAOUHSC_00581	Hypothetical protein
0 and 2 μg/mL	1,345,202	29.84722714	/	/	Non-coding region
0 and 2 μg/mL	1,450,437	27.38183709	T < - > M	SAOUHSC_01495	Hypothetical protein
0 and 2 μg/mL	1,782,559	29.43680753	S < - > G	SAOUHSC_01873	Hypothetical protein
0 and 2 μg/mL	2,062,927	27.96740905	I < - > I	SAOUHSC_02214	Conserved hypothetical phage protein
0 and 2 μg/mL	2,090,129	28.32154031	K < - > K	SAOUHSC_02257	Hypothetical protein
0 and 2 μg/mL	2,154,316	38.59800814	/	/	Non-coding region
0 and 2 μg/mL	2,537,906	29.41470633	F < - > Y	SAOUHSC_02761	Hypothetical protein
0 and 4 μg/mL	31,826	35.30010836	G < - > G	SAOUHSC_00025	GRAM_POS_ANCHORING domain-containing protein
0 and 4 μg/mL	32,318	34.98615466	N < - > N	SAOUHSC_00025	GRAM_POS_ANCHORING domain-containing protein
0 and 4 μg/mL	92,210	42.67288375	P < - > P	SAOUHSC_00085	Hypothetical protein
0 and 4 μg/mL	183,439	34.79714436	A < - > V	SAOUHSC_00169	Peptide ABC transporter permease
0 and 4 μg/mL	183,485	36.22957857	Y < - > Y	SAOUHSC_00169	Peptide ABC transporter permease
0 and 4 μg/mL	229,384	46.72291229	/	/	Non-coding region
0 and 4 μg/mL	454,194	57.69963421	/	/	Non-coding region
0 and 4 μg/mL	1,928,590	52.14833686	G < - > G	SAOUHSC_02023	Bifunctional autolysin
0 and 4 μg/mL	1,937,407	58.19686122	I < - > I	SAOUHSC_02031	Conserved hypothetical phage protein
0 and 4 μg/mL	1,938,367	50.89188388	V < - > V	SAOUHSC_02033	Phage tape measure protein
0 and 4 μg/mL	1,942,424	39.32770745	D < - > D	SAOUHSC_02036	Phage structural protein
0 and 4 μg/mL	2,446,641	38.11067526	I < - > I	SAOUHSC_02663	Hypothetical protein
0 and 4 μg/mL	2,644,158	35.68225792	F < - > F	SAOUHSC_02871	Acetyltransferase
0 and 6 μg/mL	31,826	51.06541714	G < - > G	SAOUHSC_00025	GRAM_POS_ANCHORING domain-containing protein
0 and 6 μg/mL	32,318	44.26767216	N < - > N	SAOUHSC_00025	GRAM_POS_ANCHORING domain-containing protein
0 and 6 μg/mL	89,719	46.52201906	E < - > E	SAOUHSC_00082	Hypothetical protein
0 and 6 μg/mL	92,210	64.00344237	P < - > P	SAOUHSC_00085	Hypothetical protein
0 and 6 μg/mL	94,818	43.88209971	C < - > C	SAOUHSC_00089	Hypothetical protein
0 and 6 μg/mL	183,439	41.59134039	A < - > V	SAOUHSC_00169	Peptide ABC transporter permease
0 and 6 μg/mL	183,485	54.59803224	Y < - > Y	SAOUHSC_00169	Peptide ABC transporter permease
0 and 6 μg/mL	183,488	41.30398699	A < - > A	SAOUHSC_00169	Peptide ABC transporter permease
0 and 6 μg/mL	229,384	65.77372072	/	/	Non-coding region
0 and 6 μg/mL	306,103	44.68014295	I < - > I	SAOUHSC_00293	Hypothetical protein
0 and 6 μg/mL	454,194	47.07477546	/	/	Non-coding region
0 and 6 μg/mL	768,340	46.38076319	T < - > A	SAOUHSC_00786	Hypothetical protein
0 and 6 μg/mL	1,928,590	53.5288025	G < - > G	SAOUHSC_02023	Bifunctional autolysin
0 and 6 μg/mL	1,937,407	48.04663063	I < - > I	SAOUHSC_02031	Conserved hypothetical phage protein
0 and 6 μg/mL	1,942,424	42.77951112	D < - > D	SAOUHSC_02036	Phage structural protein
0 and 6 μg/mL	2,644,158	51.38237408	F < - > F	SAOUHSC_02871	Acetyltransferase

### Genotype–Phenotype Variation Analysis of Growth Parameters vs. Significant SNPs

**FIGURE 4:**
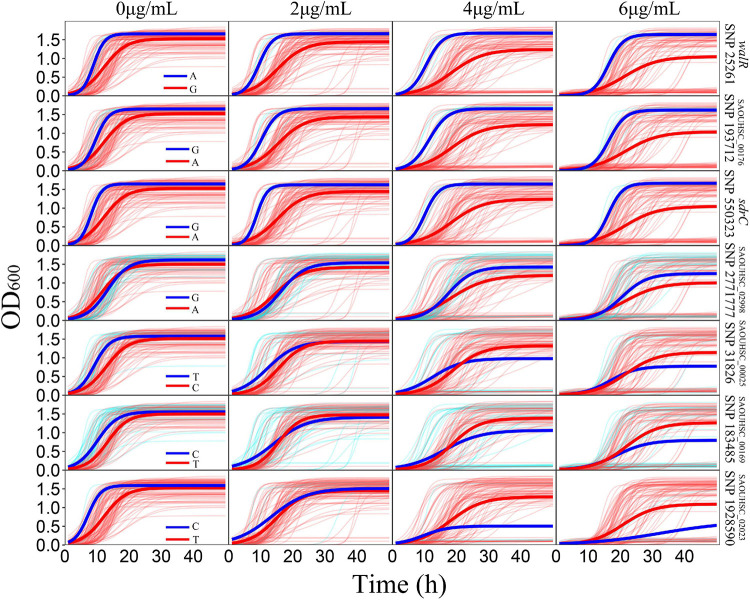
Growth variations of significant SNPs. For each SNP, strains were divided into two groups according to their genotypes. The thick red and blue lines represent the average growth curves of the two genotypes, while the thin lines in the background represent the individual growth curves.

For the genotype–phenotype variation analysis, we selected seven key genes affecting the phenotypic plasticity of *S. aureus* under various vancomycin concentrations based on their *P*-values and occurrence frequencies from functional mapping and bivariate functional mapping. The growth curves of two genotypes corresponding to the seven selected genes were fitted (Figure 4), and the parameters of the curve were assessed by *t*-tests to explore the specific effects of the genes on phenotypic plasticity (Table 3). We found that there were significant differences in the growth curves of the representative genotypes.

**TABLE 3:** Growth parameters *t*-test of two genotypes in 6 μg/mL.

Position	Gene ID	*P*-value of parameter A	*P*-value of parameter R	*P*-value of parameter λ
25,261	SAOUHSC_00020	2.81E-11	2.46E-02	5.94E-01
193,712	SAOUHSC_00176	3.88E-11	2.23E-02	4.28E-01
550,323	SAOUHSC_00544	5.82E-13	2.65E-02	3.56E-01
2,771,777	SAOUHSC_02998	1.41E-01	5.49E-02	4.52E-01
31,826	SAOUHSC_00025	7.56E-02	1.76E-01	2.22E-01
183,485	SAOUHSC_00169	4.84E-04	5.83E-03	3.08E-02
1,928,590	SAOUHSC_02023	1.49E-01	3.23E-04	9.62E-02

The SAOUHSC_00020 (SNP25261) gene was divided into two groups according to base A and G, and the growth of strains with genotype A was significantly higher than that of individuals with genotype G; the difference in growth rates between the two genotypes increased with the vancomycin concentration. A similar pattern was observed for SAOUHSC_00544 and SAOUHSC_00176. For the SAOUHSC_02998, SAOUHSC_00025, SAOUHSC_00169, and SAOUHSC_02023 genes, significant differences in growth between the two representative genotypes were present only at high vancomycin concentrations. We therefore categorized the first gene group as global regulator plasticity genes and the second group as stress response plasticity genes.

The SAOUHSC_00020 gene (containing SNP 25261) significant in three concentrations (0, 4, and 6 μg/mL) of function mapping and is therefore likely to represent a key gene affecting *S. aureus* growth. The degree of growth and maximum specific growth rate were significantly different between the two SAOUHSC_00020 genotypes examined, while there was no significant difference in the lag phase length. SAOUHSC_00176 (containing SNP 193712), which encodes a protein involved in extracellular solute binding, was significant in two sets of functional mapping (0 and 4 μg/mL); all growth parameters were significantly affected by SAOUHSC_00176. SAOUHSC_00544 (containing SNP 550323) encodes the fibrinogen-binding protein SdrC, which plays an important role in adhesion and pathogenesis. SAOUHSC_00176 also affected the entire growth process in all directions. The SAOUHSC_02998 gene (containing SNP 2771777) encodes the capsular polysaccharide biosynthesis protein Cap5C and can trigger an increase in growth rate. The SAOUHSC_00025 gene (containing SNP 31826) encodes GRAM_POS_ANCHORING domain-containing protein, SAOUHSC_00169 (containing SNP 183485) encodes an ABC transporter permease, and SAOUHSC_02023 encodes a bifunctional autolysin; these three genes are key regulators of phenotypic plasticity, as indicated by significant differences in all growth parameters at a vancomycin concentration of 6 μg/mL.

## Discussion

Phenotypic plasticity enables microorganisms to grow and develop in different environments, playing an important role in the competition and evolution of microorganisms. However, studies investigating phenotypic plasticity in microorganisms are limited (Zheng et al., 2020). In our previous work, we used a bivariate GWAS method to locate genes that affected the phenotypic plasticity related to the growth of *S. aureus* at a single time point (Rong et al., 2019). However, this was not sufficient to evaluate the entire complex bacterial growth process. In this study, we analyzed the phenotypic plasticity of 99 *S. aureus* strains grown under different antibiotic concentrations using functional mapping and bivariate functional mapping. We identified seven genes that were significantly related to growth-related phenotypic plasticity under the pressure of vancomycin in *S. aureus*.

With the increasing use of antibiotics, more drug-resistant *S. aureus* strains are emerging, posing a serious threat to human public health (Laupland, 2013). Vancomycin resistant *S. aureus* (VRSA) can adapt to antibiotic environments (Gardete and Tomasz, 2014) via cell wall thickening, reducing intracellular toxicity, reducing peptidoglycan cross-linking and modifying autolysis rates. Previous studies indicated that spontaneous mutations could significantly affect the evolution of vancomycin intermediate resistant S. *aureus* (VISA) (Chen et al., 2014; Hu et al., 2016). Phenotypic plasticity plays an important role in bacterial adaptive evolution, which highlights the importance of investigating the mechanisms driving phenotypic plasticity in *S. aureus* during vancomycin exposure.

So far, the majority of GWAS studies have explored the relationships of genes with bacterial growth and development by analyzing a single phenotype. Applying bivariate GWAS improved the scope of this method and reduced the rate of false positives (Bedo et al., 2014); however, both GWAS and bivariate GWAS are performed using a single time point. As bacterial development is a dynamic process, bacterial phenotypic plasticity studies should take into consideration the whole growth process. GWAS cannot be used to fully and accurately determine the relationship between genes and phenotypes throughout the development process (Wang et al., 2014). Our functional mapping technique based on dynamic time series data showed great potential for future applications in plasticity research.

In this study, 99 *S. aureus* strains were cultured under varying vancomycin concentrations, and 78 significant loci were identified by functional mapping and bivariate functional mapping. Seven genes that play significant roles in *S. aureus* phenotypic plasticity were identified: SAOUHSC_00020(*walR*), SAOUHSC_00176, SAOUHSC_00544(*sdrC*), SAOUHSC_02998, SAOUHSC_00025, SAOUHSC_00169, and SAOUHSC_02023. *WalR* plays a role in the development of bacterial drug resistance (Baseri et al., 2021; Zhu et al., 2021) and regulates genes involved in cell wall metabolism, virulence regulation, biofilm production, oxidative stress resistance and antibiotic resistance via the direct or indirect regulation of autolysins (Kan et al., 2007; Delaune et al., 2012; Poupel et al., 2016). Single nucleotide substitutions within the *walR* gene lead to vancomycin and daptomycin co-resistance and cause the typical cell wall thickening observed in antibiotic-resistant clinical isolates (Howden et al., 2011). SAOUHSC_00176 is involved in ATP transport and cell membrane synthesis, playing a crucial role in *S. aureus* colonization and growth (Oiki et al., 2019). SdrC is a cell surface-associated calcium-binding protein that regulates adhesion and pathogenesis and can promote bacterial adhesion by mediating interactions with components of the extracellular matrix (Batool et al., 2021; Wang et al., 2021). The SAOUHSC_02998 gene (containing SNP 2771777) encodes a protein involved in the biosynthesis of capsular polysaccharides and plays a key role in resisting adverse environments and enhancing cellular adhesion (Liu et al., 2018). SAOUHSC_00169 (SNP 183485) encodes ABC transporter permease, which is involved in the transportation of ATP and in drug resistance. An important mechanism driving bacterial antibiotic resistance is the reduction of intracellular antibiotic concentrations by multi-drug resistance proteins (Baltes et al., 2020; Gao et al., 2020). SAOUHSC_02023 (SNP 1928590) encodes bifunctional autolysin, which regulates cell division, daughter cell separation, and autolysis (Albrecht et al., 2012; Kluj et al., 2018). SAOUHSC_00025 (SNP 31826) encodes an uncharacterized GRAM_POS_ANCHORING domain-containing protein that was identified to play an important role in phenotypic plasticity regulation by our bivariate functional mapping model. The correlation between MIC and growth inhibition of each strain under vancomycin pressure was also analyzed, but not statistically significant (*P* = 0.12) (data not shown). These results indicated dynamic gene mapping techniques are more accurate and powerful.

Our genotype–phenotype growth variation analysis enabled categorization of the seven significant SNPs into two groups: global regulator genes and stress response plasticity genes. For the global regulator genes (SAOUHSC_00020, SAOUHSC_00544, and SAOUHSC_00176), one genotype exhibited superior growth traits under all vancomycin concentrations (Figure 4). For the stress response plasticity genes (SAOUHSC_02998, SAOUHSC_00025, SAOUHSC_00169, and SAOUHSC_02023), the difference in growth between the two genotypes was significant only under high vancomycin concentrations, at which one phenotype exhibited a significant growth advantage and improved plasticity (Figure 4). At the same time, the association between genotypes and MICs of these seven genes was analyzed. The result showed the genotypes of three genes (*walR*, SAOUHSC_00176, and *sdrC*) were associated with MICs, while the other four had no significant correlation (Supplementary Figure 1).

In this study, we analyzed the phenotypic plasticity of *S. aureus* under vancomycin pressure by functional mapping, which led to the identification of seven significant plasticity genes. Functional mapping and bivariate functional mapping provide novel strategies for the study of bacterial phenotypic plasticity, enabling analysis of the relationships between multiple phenotypes and genotypes throughout the dynamic development process. Our method facilitated the location of significant genes, which would not have been possible using a standard, static GWAS methodology.

## Data Availability Statement

The datasets presented in this study can be found in online repositories. The name of the repository can be found below and the accession numbers can be found in the Supplementary Material: NCBI repository, https://www.ncbi.nlm.nih.gov/Traces/study/?acc=PRJNA722566.

## Author Contributions

YJ and RW conceived and designed the experiments. DY and XZ performed the experiments. LJ, MY, and XH analyzed the data. DY and YJ wrote the manuscript. All authors reviewed the manuscript.

## Conflict of Interest

The authors declare that the research was conducted in the absence of any commercial or financial relationships that could be construed as a potential conflict of interest.

## Publisher’s Note

All claims expressed in this article are solely those of the authors and do not necessarily represent those of their affiliated organizations, or those of the publisher, the editors and the reviewers. Any product that may be evaluated in this article, or claim that may be made by its manufacturer, is not guaranteed or endorsed by the publisher.

## Footnotes

1
https://www.ncbi.nlm.nih.gov/Traces/study/?acc=PRJNA722566
