# Supplementary material for: Functional Mapping of Phenotypic Plasticity of Staphylococcus aureus Under Vancomycin Pressure
Source: Front Microbiol. 2021 Sep 9;12:696730. doi: 10.3389/fmicb.2021.696730 (PMC8458881; doi:10.3389/fmicb.2021.696730)
Supplement: Supplementary file 1 [file Data_Sheet_1.PDF]

Table S1. The source of strains

Strain number[a]	Vancomycin MIC (μg/mL)[b]	Accession number	Source of parental strain
S1 (S_p1)	10 (1)	SRX10649650	CICC(21676)
S2 (S_p2)	3 (1.5)	SRX10649651	CICC(21600 (ATCC 27217))
S3 (S_p3)	10 (1.5)	SRX10649662	ACCC(01334)
S4 (S_p4)	3 (1)	SRX10649673	ACCC(01340)
S5 (S_p5)	4 (1.5)	SRX10649684	ACCC(01332)
S6 (S_p6)	3 (1)	SRX10649695	ACCC(01331)
S7 (S_p7)	10 (1)	SRX10649706	ACCC(01339)
S8 (S_p8)	16 (1.5)	SRX10649717	CFCC(10341)
S9 (S_p9)	12 (1)	SRX10649728	CGMCC(1.8721 (ATCC 29213))
S11 (S_p11)	8 (1.5)	SRX10649739	CGMCC(1.1476)
S12 (S_p12)	3 (1)	SRX10649652	CPCC(141396)
S13 (S_p13)	4 (1)	SRX10649653	CPCC(140594)
S14 (S_p14)	3 (1)	SRX10649654	CPCC(140575)
S15 (S_p15)	16 (1.5)	SRX10649655	CICC(21648)
S16 (S_p16)	16 (1)	SRX10649656	CICC(10786)
S17 (S_p17)	4 (1.5)	SRX10649657	CICC(22942)
S18 (S_p18)	3 (1)	SRX10649658	CCTCC(AB 94004)
S19 (S_p19)	4 (1)	SRX10649659	CCTCC(AB 91093)
S20 (S_p20)	4 (1)	SRX10649660	CCTCC(AB 91053)
S21 (S_p21)	4 (1)	SRX10649661	CICC(23699)
S22 (S_p22)	3 (1.5)	SRX10649663	CICC(23656 (ATCC 25923))
S23 (S_p23)	3 (1)	SRX10649664	CICC(22944)
S24 (S_p24)	3 (1)	SRX10649665	CGMCC(1.2465 (ATCC 6538))
S25 (S_p25)	4 (1.5)	SRX10649666	CCTCC(AB 91119)
S26 (S_p26)	4 (1.5)	SRX10649667	CICC(10201)
S27 (S_p27)	8 (1)	SRX10649668	ACCC(10499 (ATCC 12600))
S28 (S_p28)	14 (1.5)	SRX10649669	ACCC(01012)
S29 (S_p29)	3 (1)	SRX10649670	CPCC(141405)
S30 (S_p30)	3 (1.5)	SRX10649671	CMCC(26003)
S31 (S_p31)	4 (1.5)	SRX10649672	CMCC(26112)
S32 (S_p32)	12 (1)	SRX10649674	ACCC(01011)

S33 (S_p33)	3 (1)	SRX10649675	CMCC(26001)
S34 (S_p34)	6 (1.5)	SRX10649676	CPCC(141431)
S35 (S_p35)	12 (1)	SRX10649677	CPCC(140660)
S36 (S_p36)	3 (1)	SRX10649678	CGMCC(1.1529)
S40 (S_p40)	8 (1.5)	SRX10649679	CAU(P1)
S41 (S_p41)	8 (1)	SRX10649680	CAU(AB18)
S42 (S_p42)	8 (1)	SRX10649681	CAU(CD1)
S43 (S_p43)	8 (1.5)	SRX10649682	CAU(CD9)
S44 (S_p44)	16 (1)	SRX10649683	CAU(CD7)
S45 (S_p45)	4 (1)	SRX10649685	ACCC(01336)
S47 (S_p47)	4 (1)	SRX10649686	BCYH
S48 (S_p48)	2 (1)	SRX10649687	BCYH
S49 (S_p49)	2.5 (1)	SRX10649688	BCYH
S50 (S_p50)	3 (1.5)	SRX10649689	BCYH
S51 (S_p51)	12 (1)	SRX10649690	BCYH
S52 (S_p52)	5 (1)	SRX10649691	BCYH
S53 (S_p53)	3.5 (1)	SRX10649692	BCYH
S54 (S_p54)	2.5 (1)	SRX10649693	BCYH
S55 (S_p55)	6 (1.5)	SRX10649694	BCYH
S56 (S_p56)	7 (1)	SRX10649696	BCYH
S57 (S_p57)	18 (1)	SRX10649697	BCYH
S58 (S_p58)	5 (1.5)	SRX10649698	BCYH
S59 (S_p59)	6 (1)	SRX10649699	BCYH
S60 (S_p60)	8 (1)	SRX10649700	BCYH
S61 (S_p61)	3 (1)	SRX10649701	BCYH
S62 (S_p62)	8 (1)	SRX10649702	BCYH
S63 (S_p63)	4 (1)	SRX10649703	BCYH
S64 (S_p64)	6 (1.5)	SRX10649704	BCYH
S65 (S_p65)	4 (1)	SRX10649705	BCYH
S66 (S_p66)	7 (1)	SRX10649707	BCYH
S67 (S_p67)	7 (1)	SRX10649708	BCYH
S68 (S_p68)	4.5 (1)	SRX10649709	BCYH
S69 (S_p69)	8 (1.5)	SRX10649710	BCYH
S70 (S_p70)	6 (1)	SRX10649711	BCYH

S71 (S_p71)	6 (1.5)	SRX10649712	BCYH
S72 (S_p72)	5 (1)	SRX10649713	BCYH
S73 (S_p73)	2 (1)	SRX10649714	BCYH
S74 (S_p74)	18 (1)	SRX10649715	BCYH
S75 (S_p75)	3 (1)	SRX10649716	BCYH
S76 (S_p76)	3 (1.5)	SRX10649718	BCYH
S77 (S_p77)	3 (1)	SRX10649719	BCYH
S78 (S_p78)	8 (1)	SRX10649720	BCYH
S79 (S_p79)	8 (1.5)	SRX10649721	BCYH
S80 (S_p80)	3 (1)	SRX10649722	BCYH
S81 (S_p81)	4 (1)	SRX10649723	BCYH
S82 (S_p82)	3 (1)	SRX10649724	BCYH
S83 (S_p83)	12 (1)	SRX10649725	BCYH
S84 (S_p84)	9 (1.5)	SRX10649726	BCYH
S85 (S_p85)	8 (1.5)	SRX10649727	BCYH
S87 (S_p87)	8 (1.5)	SRX10649729	BCYH
S88 (S_p88)	2.5 (1)	SRX10649730	BCYH
S89 (S_p89)	6 (1)	SRX10649731	BCYH
S90 (S_p90)	6 (1)	SRX10649732	BCYH
S91 (S_p91)	6 (1)	SRX10649733	BCYH
S92 (S_p92)	2 (1)	SRX10649734	BCYH
S93 (S_p93)	2 (1.5)	SRX10649735	BCYH
S94 (S_p94)	3.5 (1)	SRX10649736	BCYH
S95 (S_p95)	2.5 (1)	SRX10649737	BCYH
S96 (S_p96)	2 (1)	SRX10649738	BCYH
S97 (S_p97)	6 (1.5)	SRX10649740	BCYH
S98 (S_p98)	4 (1.5)	SRX10649741	BCYH
S99 (S_p99)	5 (1)	SRX10649742	BCYH
S100 (S_p100)	6 (1.5)	SRX10649743	BCYH
S101 (S_p101)	16 (1)	SRX10649744	BCYH
S102 (S_p102)	4.5 (1)	SRX10649745	BCYH
S103 (S_p103)	4 (1)	SRX10649746	BCYH
S104 (S_p104)	2 (1)	SRX10649747	BCYH
S105 (S_p105)	2 (1)	SRX10649748	BCYH

[a]: Strain number (parental strain number);

[b]: MIC of treated strain (MIC of parental strain).

CGMCC: China Common Microbial Culture Collection

CFCC: China Forestry Microbial Culture Collection Management Center

CICC: China Industrial Microbial Culture Collection Management Center

CMCC: China Medical Microbial Culture Collection Management Center

ACCC: China Agricultural Culture Collection Center

CCTCC: China Type Culture Collection

CPCC: China Pharmaceutical Microbial Culture Collection Management Center

CAU: China Agricultural University School of Veterinary Medicine

BCYH: Beijing Chaoyang Hospital

Table S2. Sequencing Data Quality Statistics

Sample	Raw Base(bp)	Clean Base(bp)	Effective Rate(%)	Error Rate(%)	Q20(%)	Q30(%)	GC Content(%)
S1	1,276,220,100	1,234,355,700	96.72	0.01	97.34	93.47	32.76
S2	1,207,527,600	1,164,902,400	96.47	0.01	97.34	93.45	32.58
S3	1,097,338,500	1,054,156,500	96.06	0.01	97.24	93.25	32.95
S4	1,024,674,000	996,435,000	97.24	0.01	97.58	93.9	32.89
S5	1,005,031,500	947,010,600	94.23	0.01	96.06	90.91	33.4
S6	982,932,600	941,070,000	95.74	0.01	96.9	92.61	32.8
S7	1,027,182,000	977,821,200	95.19	0.01	96.73	92.27	33.28
S8	1,158,221,700	1,097,237,700	94.73	0.02	96.34	91.58	33.14
S9	1,237,908,900	1,170,174,300	94.53	0.02	96.24	91.35	32.33
S11	1,080,319,800	1,062,825,600	98.38	0.01	97.88	94.43	32.62
S12	1,331,588,100	1,311,063,600	98.46	0.01	97.98	94.64	32.78
S13	1,098,426,600	1,080,812,100	98.4	0.01	97.99	94.65	32.93
S14	977,382,900	960,207,600	98.24	0.01	98.04	94.77	32.77
S15	1,153,930,800	1,132,962,300	98.18	0.01	98.06	94.8	32.73
S16	1,292,799,000	1,267,027,200	98.01	0.01	98.1	94.92	32.75
S17	1,103,493,900	1,083,543,900	98.19	0.01	98.06	94.83	32.83
S18	1,101,992,400	1,069,692,900	97.07	0.01	97.96	94.6	33.14
S19	1,172,319,900	1,128,285,000	96.24	0.01	97	92.72	32.87
S20	2,582,253,600	2,508,086,700	97.13	0.01	97.91	94.82	33.37
S21	1,154,775,300	1,114,807,200	96.54	0.01	97.9	94.45	33.32
S22	1,005,323,100	944,565,900	93.96	0.02	95.95	90.75	33.28
S23	1,417,425,600	1,363,396,500	96.19	0.01	96.32	91.38	32.88
S24	2,658,411,600	2,581,080,000	97.09	0.01	97.84	94.66	33.35
S25	1,187,688,600	1,141,178,100	96.08	0.01	97.14	93.05	33.09
S26	1,088,412,900	1,053,000,300	96.75	0.01	97.22	93.23	33.21
S27	1,427,494,500	1,378,908,300	96.6	0.01	96.51	91.74	32.92
S28	1,184,597,400	1,140,968,400	96.32	0.01	96.94	92.67	33.02
S29	1,216,412,700	1,173,562,200	96.48	0.01	97.19	93.14	32.81
S30	1,055,945,100	997,863,000	94.5	0.02	96.28	91.43	33.39
S31	1,103,836,500	1,049,487,600	95.08	0.01	96.51	91.85	32.7
S32	1,094,899,500	1,054,940,400	96.35	0.01	97.13	93.06	33.15
S33	988,878,600	948,252,600	95.89	0.01	96.84	92.44	33.06
S34	1,087,321,200	1,064,582,100	97.91	0.01	97.63	93.98	32.57
S35	1,220,067,000	1,192,517,100	97.74	0.01	97.56	93.84	32.62
S36	1,033,618,500	982,873,500	95.09	0.01	96.63	92.11	32.81
S37	2,619,620,700	2,332,630,800	89.04	0.01	97.18	93.37	33.42
S38	1,001,803,800	949,707,300	94.8	0.01	96.53	91.9	32.95
S39	1,032,318,900	972,514,800	94.21	0.02	96.56	92.03	33.2
S40	1,143,993,600	1,093,744,200	95.61	0.01	96.8	92.39	33.09

S41	1,254,948,600	1,209,438,900	96.37	0.01	97.13	93.06	33.06
S42	991,215,300	960,145,800	96.87	0.01	97.37	93.5	32.25
S47	992,943,200	991,596,400	99.86	0.02	95.92	89.57	32.79
S48	1,393,589,000	1,392,274,600	99.91	0.02	98.18	94.31	32.95
S49	1,290,140,000	1,289,000,800	99.91	0.02	98.22	94.39	33.00
S50	1,411,012,000	1,409,713,000	99.91	0.02	98.25	94.47	32.57
S51	1,216,390,000	1,214,814,000	99.87	0.02	97.88	93.60	33.02
S52	744,378,600	743,573,600	99.89	0.02	96.39	90.47	32.59
S53	1,145,201,800	1,144,108,800	99.90	0.02	98.34	94.71	32.97
S54	1,415,867,400	1,414,362,600	99.89	0.02	98.07	94.03	32.61
S55	1,176,410,200	1,175,261,400	99.90	0.02	98.05	94.00	32.99
S56	1,168,824,600	1,168,088,200	99.94	0.02	98.24	94.49	32.95
S57	983,217,200	982,153,400	99.89	0.02	96.34	90.37	32.87
S58	1,371,531,400	1,370,453,400	99.92	0.02	98.35	94.73	33.34
S59	1,227,171,200	1,226,070,200	99.91	0.02	98.38	94.78	32.97
S60	1,262,973,000	1,261,771,200	99.90	0.02	98.23	94.44	33.14
S61	939,894,200	938,632,200	99.87	0.02	95.92	89.59	32.55
S62	1,126,057,000	1,125,068,600	99.91	0.02	98.39	94.84	32.95
S63	1,383,463,400	1,382,139,400	99.90	0.02	98.31	94.63	33.11
S64	1,186,343,600	1,185,144,400	99.90	0.02	98.36	94.79	32.92
S65	915,056,000	914,197,400	99.91	0.02	98.34	94.72	32.95
S66	1,275,720,400	1,274,495,600	99.90	0.02	98.27	94.53	32.87
S67	1,318,738,200	1,317,417,600	99.90	0.02	98.05	93.97	32.91
S68	1,026,778,600	1,025,819,000	99.91	0.02	98.28	94.55	32.86
S69	1,573,492,400	1,571,885,400	99.90	0.02	98.27	94.53	32.92
S70	1,174,190,800	1,173,100,400	99.91	0.02	98.16	94.22	32.59
S71	1,289,906,000	1,288,645,400	99.90	0.02	98.12	94.18	32.99
S72	1,206,277,600	1,205,180,600	99.91	0.02	98.20	94.34	32.86
S73	1,216,988,600	1,215,840,200	99.91	0.02	98.35	94.78	32.78
S74	1,198,695,600	1,197,631,200	99.91	0.02	98.35	94.78	33.19
S75	807,886,200	807,119,600	99.91	0.02	96.61	90.86	32.66
S76	863,705,000	862,667,200	99.88	0.02	96.17	90.06	32.55
S77	1,089,809,800	1,088,782,000	99.91	0.02	98.29	94.62	32.99
S78	1,187,248,800	1,185,984,400	99.89	0.02	98.25	94.53	32.70
S79	1,335,622,800	1,334,486,400	99.91	0.02	98.32	94.66	33.02
S80	863,216,400	862,345,000	99.90	0.02	98.03	93.94	32.88
S81	930,890,000	929,796,000	99.88	0.02	96.36	90.43	32.77
S82	1,142,089,600	1,141,110,200	99.91	0.02	98.28	94.55	32.97
S83	1,072,407,400	1,071,142,400	99.88	0.02	97.35	92.35	33.12
S84	1,093,385,000	1,092,370,000	99.91	0.02	98.35	94.76	32.84
S85	1,252,415,600	1,251,139,200	99.90	0.02	98.19	94.38	33.14
S87	1,164,049,800	1,162,950,200	99.91	0.02	98.06	94.03	32.84
S88	1,065,622,800	1,064,656,600	99.91	0.02	98.18	94.30	33.00
S89	1,265,457,000	1,264,197,800	99.90	0.02	98.29	94.61	32.89

S90	1,035,089,400	1,034,180,600	99.91	0.02	98.25	94.48	32.91
S91	923,461,800	922,600,000	99.91	0.02	96.41	90.43	32.61
S92	1,056,494,800	1,055,237,400	99.88	0.02	96.29	90.28	32.67
S93	1,185,983,000	1,184,760,600	99.90	0.02	98.14	94.25	33.00
S94	1,217,911,800	1,216,676,800	99.90	0.02	98.19	94.35	32.86
S95	792,535,000	791,590,400	99.88	0.02	96.25	90.19	32.73
S96	1,010,366,000	1,009,447,200	99.91	0.02	98.41	94.91	32.93
S97	863,813,800	862,901,400	99.89	0.02	96.63	90.88	32.86
S98	861,060,200	860,135,200	99.89	0.02	96.53	90.75	32.55
S99	1,212,486,800	1,211,439,800	99.91	0.02	98.17	94.29	33.18
S100	1,177,699,200	1,176,636,400	99.91	0.02	98.16	94.25	33.02
S101	697,937,200	697,161,000	99.89	0.02	96.55	90.79	32.92
S102	705,441,200	704,682,600	99.89	0.02	96.35	90.37	32.76
S103	1,178,078,200	1,176,965,800	99.91	0.02	98.12	94.16	32.87
S104	1,154,885,400	1,153,944,400	99.92	0.02	98.30	94.59	33.02
S105	912,119,800	911,233,800	99.90	0.02	98.19	94.36	33.02

Table S3. Result Comparison with Reference Sequence

Sample	Mapped reads	Total reads	Mapping rate(%)	Average depth(X)	Coverage at least 1X(%)	Coverage at least 4X(%)
S1	7,382,267	8,229,038	89.71	310.88	97.34	96.5
S2	6,262,497	7,766,016	80.64	266.81	95.95	94.99
S3	5,927,812	7,027,710	84.35	266.87	94.16	91.61
S4	5,921,076	6,642,900	89.13	266.42	91.76	89.69
S5	5,448,061	6,313,404	86.29	258.01	91.42	90.98
S6	5,617,228	6,273,800	89.53	254.63	93.13	91.11
S7	5,845,224	6,518,808	89.67	260.91	94.51	91.29
S8	6,428,237	7,314,918	87.88	275.54	97.13	95.22
S9	5,966,937	7,801,162	76.49	256.82	95.97	94.26
S11	6,491,319	7,085,504	91.61	311.48	94.06	93.42
S12	8,222,586	8,740,424	94.08	380.47	93.93	93.49
S13	6,853,124	7,205,414	95.11	318.57	94.82	93.43
S14	5,991,961	6,401,384	93.6	284.91	93.9	93.46
S15	6,893,549	7,553,082	91.27	327.43	93.96	93.64
S16	7,405,335	8,446,848	87.67	338.2	93.86	93.64
S17	6,453,867	7,223,626	89.34	301.54	93.82	92.87
S18	6,507,015	7,131,286	91.25	306.39	94	93.43
S19	7,082,944	7,521,900	94.16	319.7	94.69	93.57
S20	15,989,549	16,720,578	95.63	682.25	93.97	93.51
S21	6,994,291	7,432,048	94.11	334.22	93.93	93.49
S22	5,357,872	6,297,106	85.08	240.28	95.51	91.79
S23	6,721,276	9,089,310	73.95	304.99	93.24	92.96
S24	16,117,955	17,207,200	93.67	698.14	94.01	93.55
S25	6,599,709	7,607,854	86.75	289.14	93.56	91.2
S26	6,694,558	7,020,002	95.36	290.09	94.95	93.64
S27	7,556,391	9,192,722	82.2	334.16	94.09	93.6
S28	6,599,053	7,606,456	86.76	293.69	94.62	93
S29	7,408,680	7,823,748	94.69	329.38	94.72	93.54
S30	6,236,589	6,652,420	93.75	278.06	95.42	93.59
S31	5,766,152	6,996,584	82.41	258.29	95.1	91.5
S32	6,164,318	7,032,936	87.65	270.07	94.15	91.27
S33	5,504,153	6,321,684	87.07	244.52	95.03	93.6
S34	5,695,393	7,097,214	80.25	255.23	94.34	92.7
S35	7,019,869	7,950,114	88.3	313.25	94.95	93.74
S36	6,175,491	6,552,490	94.25	279.8	95.07	91.94
S37	13,534,637	15,550,872	87.03	636.77	89.62	89.38
S38	5,179,634	6,331,382	81.81	232.42	95.15	92.14
S39	5,759,571	6,483,432	88.84	243.55	98.17	93.27
S40	5,413,764	7,291,628	74.25	239.38	95.88	93.11

S41	6,544,823	8,062,926	81.17	283.18	95.56	93.8
S42	4,601,587	6,400,972	71.89	200.03	96.47	95.01
S47	8,887,257	9,915,964	89.63	285.62	91.36	91.02
S48	12,609,733	13,922,746	90.57	379.11	94.41	93.49
S49	12,100,805	12,890,008	93.88	375.54	94.37	92.89
S50	10,231,222	14,097,130	72.58	297.97	95.30	94.86
S51	10,773,603	12,148,140	88.69	330.45	95.06	93.69
S52	6,481,361	7,435,736	87.17	203.30	94.00	93.78
S53	10,063,494	11,441,088	87.96	309.15	91.85	90.00
S54	11,820,439	14,143,626	83.57	353.00	92.62	91.91
S55	10,255,723	11,752,614	87.26	306.79	96.66	96.19
S56	10,492,827	11,680,882	89.83	339.79	94.16	93.66
S57	8,806,858	9,821,534	89.67	281.10	90.77	90.05
S58	12,268,505	13,704,534	89.52	371.69	95.23	95.08
S59	10,885,995	12,260,702	88.79	323.85	96.44	96.18
S60	11,482,111	12,617,712	91.00	348.05	95.24	95.06
S61	8,390,361	9,386,322	89.39	263.58	92.42	91.86
S62	9,863,331	11,250,686	87.67	296.77	95.07	94.67
S63	12,434,081	13,821,394	89.96	369.14	91.07	89.91
S64	11,172,632	11,851,444	94.27	341.01	94.37	93.13
S65	7,953,152	9,141,974	87.00	246.58	94.58	94.25
S66	11,130,550	12,744,956	87.33	330.18	96.38	96.19
S67	11,583,750	13,174,176	87.93	336.23	96.66	96.19
S68	8,667,673	10,258,190	84.50	258.19	95.17	94.80
S69	14,127,078	15,718,854	89.87	406.69	95.41	95.09
S70	8,596,711	11,731,004	73.28	255.31	95.57	93.95
S71	11,517,108	12,886,454	89.37	343.10	95.45	94.90
S72	11,074,369	12,051,806	91.89	334.15	92.76	92.01
S73	10,987,819	12,158,402	90.37	335.55	92.95	92.37
S74	10,690,449	11,976,312	89.26	320.28	92.13	90.06
S75	7,522,835	8,071,196	93.21	237.06	93.45	93.23
S76	7,084,637	8,626,672	82.12	221.95	94.23	93.93
S77	9,790,139	10,887,820	89.92	295.01	94.16	93.72
S78	10,084,199	11,859,844	85.03	303.32	94.18	93.93
S79	12,458,181	13,344,864	93.36	365.64	95.49	93.84
S80	7,805,529	8,623,450	90.52	235.90	94.88	93.49
S81	8,499,028	9,297,960	91.41	261.52	95.46	94.93
S82	9,990,631	11,411,102	87.55	294.12	94.31	93.51
S83	9,579,998	10,711,424	89.44	281.90	96.02	93.16
S84	9,841,577	10,923,700	90.09	299.36	94.09	93.04
S85	11,216,237	12,511,392	89.65	345.17	91.08	90.12
S87	10,333,515	11,629,502	88.86	308.68	94.01	91.44
S88	9,630,175	10,646,566	90.45	285.69	95.26	93.56
S89	11,539,094	12,641,978	91.28	354.53	92.35	91.96

S90	8,755,154	10,341,806	84.66	255.71	96.39	94.10
S91	8,048,501	9,226,000	87.24	255.54	91.52	91.04
S92	9,270,842	10,552,374	87.86	288.14	93.84	93.56
S93	10,588,129	11,847,606	89.37	314.64	96.24	95.09
S94	10,441,368	12,166,768	85.82	322.37	91.49	90.87
S95	6,882,238	7,915,904	86.94	222.62	90.94	89.70
S96	8,884,730	10,094,472	88.02	273.20	92.60	90.90
S97	7,632,016	8,629,014	88.45	237.24	95.09	94.84
S98	7,575,768	8,601,352	88.08	232.49	97.04	96.53
S99	10,589,228	12,114,398	87.41	309.70	94.99	93.74
S100	10,557,879	11,766,364	89.73	309.93	95.57	95.07
S101	6,229,103	6,971,610	89.35	201.75	90.85	90.09
S102	6,293,047	7,046,826	89.30	193.66	95.47	94.85
S103	10,424,827	11,769,658	88.57	307.69	94.70	93.63
S104	10,988,581	11,539,444	95.23	327.33	94.34	93.05
S105	8,696,878	9,112,338	95.44	265.71	94.28	93.53

Table S4. Three parameters of curve fitting

Strain number	0μg/mL			2μg/mL			4μg/mL			6μg/mL		
	A	R	λ	A	R	λ	A	R	λ	A	R	λ
S1	1.62	0.45	3.77	1.53	0.49	5.36	0.27	0.00	13.61	0.11	0.02	0.00
S2	1.50	0.21	3.26	1.46	0.22	30.35	0.15	0.01	0.00	0.10	0.01	0.00
S3	1.66	0.26	6.12	1.66	0.23	6.39	1.71	0.17	6.56	1.61	0.32	11.87
S4	1.67	0.18	6.22	1.62	0.38	37.07	0.23	0.00	2.30	0.23	0.00	21.36
S5	1.60	0.15	5.22	1.54	0.09	5.89	1.45	0.08	21.98	0.09	0.07	0.14
S6	0.78	0.04	2.66	0.97	0.03	6.23	0.12	0.01	0.00	0.09	0.02	0.28
S7	1.65	0.09	6.59	1.69	0.09	7.04	1.74	0.09	8.00	0.09	0.06	0.11
S8	1.69	0.12	6.63	1.63	0.08	5.50	1.65	0.09	8.57	1.56	0.07	11.68
S9	1.61	0.27	6.68	1.60	0.27	6.98	1.64	0.35	8.09	1.48	0.06	13.96
S11	1.60	0.26	10.30	1.58	0.15	10.62	1.64	0.12	10.64	1.56	0.22	13.73
S12	1.02	0.25	6.99	1.07	0.10	31.17	0.23	0.00	18.91	0.22	0.00	1.23
S13	1.64	0.21	4.41	1.62	0.27	5.96	1.69	0.20	8.50	1.49	0.18	23.69
S14	1.56	0.20	2.26	1.61	0.12	8.24	0.29	0.00	0.68	0.10	0.09	0.70
S15	1.66	0.24	6.79	1.65	0.18	6.90	1.71	0.18	6.81	1.62	0.17	12.74
S16	1.63	0.40	7.60	1.72	0.17	5.02	1.72	0.17	8.08	1.57	0.18	10.86
S17	1.66	0.25	2.74	1.64	0.32	3.88	0.13	0.01	0.00	0.15	0.02	0.00
S18	1.51	0.35	7.31	1.55	0.26	11.51	1.56	0.22	19.80	1.49	0.18	36.39
S19	1.65	0.11	4.90	1.40	0.03	0.27	2.07	0.04	22.27	0.26	0.00	2.67
S20	1.50	0.33	5.53	1.51	0.09	7.16	0.20	0.02	0.00	0.16	0.02	0.00
S21	1.56	0.30	6.75	1.50	0.18	8.80	1.44	0.17	19.81	0.24	0.00	2.61
S22	1.67	0.11	1.55	0.18	0.08	0.00	0.10	0.05	0.18	0.13	0.01	0.00
S23	1.59	0.19	5.26	1.61	0.16	5.63	1.66	0.15	5.57	1.63	0.15	11.38
S24	1.40	0.18	5.59	1.11	0.09	9.63	0.09	0.08	0.28	0.08	0.03	1.26
S25	1.75	0.12	3.16	0.20	0.09	0.00	0.09	0.08	0.28	0.13	0.02	0.00
S26	1.60	0.10	4.27	1.79	0.05	6.08	1.90	0.05	15.70	0.09	0.03	1.31
S27	1.39	0.08	7.50	1.24	0.08	0.00	1.15	0.07	7.76	1.09	0.07	11.10
S28	1.72	0.25	5.27	1.67	0.28	3.83	1.77	0.28	5.40	1.61	0.14	12.10
S29	1.73	0.22	5.18	1.70	0.30	8.33	1.65	0.17	24.25	0.09	0.04	1.17
S30	1.69	0.21	2.39	1.61	0.25	19.03	1.44	0.17	18.87	0.10	0.05	1.24
S31	1.62	0.12	0.22	1.62	0.11	1.72	1.43	0.19	24.12	0.18	0.03	0.00
S32	1.69	0.27	3.95	1.66	0.34	4.55	1.66	0.24	4.86	1.52	0.19	11.36
S33	1.74	0.17	6.25	1.72	0.09	13.08	0.11	0.01	0.00	0.14	0.02	0.00
S34	1.84	0.09	7.48	1.75	0.10	4.55	1.83	0.10	7.53	1.62	0.19	12.12
S35	1.66	0.32	5.56	1.61	0.34	5.94	1.62	0.30	6.34	1.68	0.25	12.15
S36	1.51	0.30	7.62	1.41	0.25	37.02	0.09	0.07	0.27	0.19	0.03	0.00
S37	1.60	0.26	4.52	1.54	0.26	5.65	1.57	0.21	7.34	1.65	0.25	14.84
S38	1.69	0.14	4.49	1.59	0.22	9.69	1.61	0.18	12.02	1.63	0.14	27.27
S39	1.65	0.15	2.46	1.60	0.18	3.25	1.65	0.16	2.69	1.59	0.14	11.65
S40	1.62	0.12	7.14	1.63	0.10	8.72	1.59	0.13	10.45	1.65	0.10	10.77
S41	1.66	0.23	3.84	1.67	0.23	4.73	1.67	0.17	3.89	1.68	0.16	11.46
S42	1.59	0.22	2.92	1.67	0.19	11.54	0.12	0.03	0.00	0.17	0.02	0.00

S47	1.57	0.11	7.32	1.56	0.09	9.35	1.60	0.08	10.74	1.72	0.09	14.84
S48	1.68	0.17	10.07	1.66	0.15	10.69	1.60	0.18	14.53	1.56	0.20	23.99
S49	1.69	0.24	6.42	1.62	0.19	15.24	0.10	0.01	0.00	0.51	0.01	12.36
S50	1.51	0.30	7.95	1.47	0.15	10.86	1.28	0.19	13.87	1.57	0.16	23.95
S51	1.47	0.13	10.30	1.39	0.08	10.79	1.39	0.07	11.85	1.55	0.09	20.60
S52	1.54	0.20	9.39	1.47	0.17	11.11	1.44	0.15	12.88	1.63	0.19	16.95
S53	1.55	0.20	5.50	1.55	0.26	6.68	1.53	0.20	6.92	1.61	0.13	13.68
S54	1.54	0.30	4.57	1.42	0.31	7.10	0.10	0.00	0.00	0.15	0.02	0.00
S55	1.72	0.26	9.69	1.75	0.31	11.51	1.76	0.24	14.51	1.78	0.29	19.21
S56	1.53	0.17	10.33	1.53	0.11	11.30	1.44	0.08	12.60	1.66	0.11	20.62
S57	1.73	0.21	10.87	1.70	0.15	11.50	1.77	0.24	13.05	1.67	0.18	12.32
S58	1.69	0.20	11.16	1.66	0.21	11.75	1.71	0.22	11.51	1.75	0.31	15.38
S59	1.73	0.26	10.02	1.73	0.25	10.41	1.79	0.25	13.65	1.69	0.24	15.47
S60	1.12	0.08	11.05	1.44	0.11	11.22	1.39	0.11	12.29	1.49	0.07	10.93
S61	1.41	0.21	5.35	1.44	0.18	8.69	1.22	0.09	28.08	0.10	0.00	0.00
S62	1.35	0.09	1.92	1.32	0.13	11.68	1.27	0.10	13.21	1.48	0.16	16.48
S63	1.35	0.23	10.32	1.31	0.19	10.56	1.30	0.12	12.10	1.47	0.10	14.24
S64	1.67	0.32	11.88	1.71	0.22	11.37	1.67	0.16	12.83	1.71	0.17	14.96
S65	1.37	0.32	8.35	1.38	0.14	8.14	1.33	0.11	9.31	1.47	0.19	14.22
S66	1.77	0.20	9.42	1.76	0.20	10.32	1.52	0.11	18.31	1.78	0.19	15.82
S67	1.62	0.25	12.15	1.72	0.18	11.65	1.64	0.18	13.55	1.61	0.24	14.91
S68	1.59	0.19	9.07	1.67	0.15	9.18	1.56	0.10	8.47	1.52	0.15	14.54
S69	1.35	0.09	13.86	1.49	0.04	11.51	1.39	0.04	14.10	1.07	0.06	13.85
S70	1.66	0.23	12.56	1.70	0.17	12.10	1.63	0.20	13.80	1.42	0.24	15.38
S71	1.33	0.13	10.56	1.40	0.12	11.37	1.37	0.13	13.46	1.50	0.14	15.61
S72	1.57	0.23	11.92	1.52	0.16	10.50	1.38	0.11	11.17	1.60	0.23	13.66
S73	1.59	0.16	10.83	1.68	0.12	10.56	1.59	0.07	15.05	1.55	0.06	18.15
S74	1.74	0.25	12.31	1.79	0.19	13.28	1.74	0.15	14.57	1.81	0.30	19.03
S75	1.59	0.35	9.08	1.64	0.27	10.94	1.65	0.19	15.68	1.72	0.21	20.09
S76	1.60	0.19	11.36	1.59	0.13	14.79	1.83	0.22	15.05	1.54	0.09	26.62
S77	1.55	0.14	8.96	1.52	0.11	9.24	1.50	0.13	12.28	0.10	0.00	0.00
S78	1.62	0.18	7.68	1.64	0.15	8.28	1.65	0.14	9.40	1.70	0.08	13.66
S79	1.42	0.22	10.42	1.38	0.28	11.72	1.36	0.23	11.82	1.32	0.22	14.86
S80	1.14	0.11	9.06	1.11	0.12	12.09	1.32	0.11	13.99	1.18	0.22	22.48
S81	1.27	0.20	5.86	1.26	0.10	6.36	1.31	0.13	7.38	1.29	0.21	15.67
S82	1.29	0.11	10.83	1.29	0.13	13.34	1.36	0.10	12.54	1.37	0.10	15.16
S83	1.48	0.20	10.34	1.47	0.15	12.07	1.47	0.13	12.04	1.60	0.25	17.77
S84	1.66	0.31	9.39	1.65	0.29	11.44	1.75	0.18	11.57	1.74	0.25	15.31
S85	1.00	0.06	11.06	0.83	0.06	13.41	1.08	0.05	11.22	0.89	0.08	10.59
S87	1.36	0.18	9.57	1.36	0.11	10.06	1.36	0.17	13.50	1.49	0.19	14.50
S88	1.06	0.06	11.09	0.99	0.06	12.65	1.06	0.04	14.88	0.03	0.01	59.32
S89	1.71	0.16	8.49	1.71	0.16	9.07	1.68	0.18	10.46	1.69	0.17	12.21
S90	1.33	0.09	11.39	1.38	0.10	11.24	1.23	0.07	10.30	1.45	0.12	14.40
S91	1.16	0.13	8.10	1.13	0.10	8.77	1.21	0.11	14.83	0.09	0.00	0.00

S92	1.47	0.16	8.90	1.41	0.17	9.96	1.41	0.13	11.65	1.36	0.20	18.67
S93	1.36	0.15	7.90	1.51	0.16	14.92	1.52	0.17	22.36	1.61	0.16	23.88
S94	1.34	0.10	4.64	1.38	0.10	7.73	1.38	0.11	24.79	0.10	0.00	0.00
S95	1.37	0.20	6.94	1.58	0.08	12.25	0.10	0.00	0.00	0.10	0.01	0.00
S96	1.30	0.18	4.45	0.10	0.00	0.00	0.10	0.00	0.00	0.35	0.00	2.06
S97	1.25	0.08	4.52	1.28	0.27	15.65	1.25	0.25	19.29	1.32	0.17	21.89
S98	1.57	0.15	9.99	1.53	0.15	11.81	1.59	0.13	13.47	1.32	0.14	24.57
S99	1.58	0.35	8.55	1.57	0.45	9.64	1.60	0.25	11.24	1.60	0.25	14.68
S100	1.19	0.06	6.14	1.24	0.10	9.74	1.11	0.09	15.95	1.58	0.18	34.90
S101	1.79	0.21	11.19	1.79	0.21	11.66	1.74	0.20	12.47	1.63	0.16	14.06
S102	1.15	0.13	9.91	1.30	0.21	11.61	1.27	0.22	15.25	1.33	0.24	18.91
S103	1.53	0.13	6.51	1.59	0.11	7.21	1.59	0.09	8.97	0.36	0.00	3.66
S104	1.23	0.12	6.02	1.29	0.11	9.76	1.32	0.09	21.25	1.34	0.16	35.87
S105	1.13	0.10	9.53	1.17	0.07	12.08	1.19	0.06	16.30	1.34	0.12	25.56

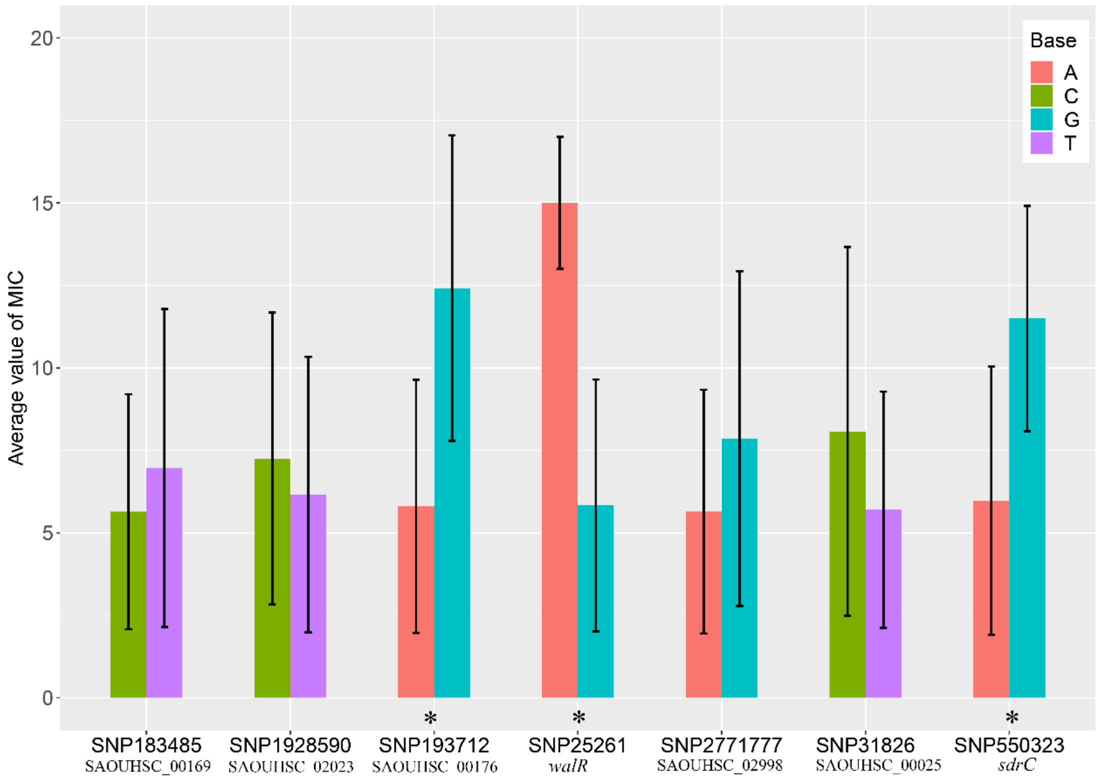

Figure S1: Genotypes and MIC association analysis diagram.
The y-axis represents the mean value of MIC, and the x-axis represents selected genes. The columns with different colors represent the mean MIC of the strain corresponding to different bases. * Indicates statistically significant of association analysis.
